# Can Parameters Other than Minimal Axial Diameter in MRI and PET/CT Further Improve Diagnostic Accuracy for Equivocal Retropharyngeal Lymph Nodes in Nasopharyngeal Carcinoma?

**DOI:** 10.1371/journal.pone.0163741

**Published:** 2016-10-13

**Authors:** Yu-Wen Wang, Chin-Shun Wu, Guo-Yi Zhang, Chih-Han Chang, Kuo-Sheng Cheng, Wei-Jen Yao, Yu-Kang Chang, Tsair-Wei Chien, Li-Ching Lin, Keng-Ren Lin

**Affiliations:** 1 Department of Biomedical Engineering, National Cheng Kung University, Tainan, Taiwan; 2 Department of Radiation Oncology, Chi Mei Medical Center, Liouying, Tainan, Taiwan; 3 Department of Nursing, Min-Hwei Junior College of Health Care Management, Tainan, Taiwan; 4 Division of Nuclear Medicine, Department of Medical Imaging, Chi Mei Medical Center, Liouying, Tainan, Taiwan; 5 Cancer Center, Foshan Hospital, Sun Yat-sen University, Foshan, People’s Republic of China; 6 Department of Nuclear Medicine, National Cheng Kung University Hospital, Tainan, Taiwan; 7 Department of Medical Imaging, Chi Mei Medical Center, Liouying, Tainan, Taiwan; 8 Department of Medical research, Chi-Mei Medical Center, Tainan, Taiwan; 9 Department of Radiation Oncology, Chi-Mei Foundation Medical Center, Tainan, Taiwan; 10 Department of Optometry, Chung Hwa University of Medical Technology, Tainan, Taiwan; 11 Medical Device Innovation Center, National Cheng Kung University, Tainan, Taiwan; Institute of Biomedical Sciences, TAIWAN

## Abstract

**Purpose:**

Minimal axial diameter (MIAD) in magnetic resonance imaging (MRI) was recognized as the most useful parameter in diagnosing lateral retropharyngeal lymph (LRPL) nodes in nasopharyngeal carcinoma (NPC). This study aims to explore the additional nodal parameters in MRI and positron emission tomography–computed tomography for increasing the prediction accuracy.

**Materials and Methods:**

A total of 663 LRPL nodes were retrospectively collected from 335 patients with NPC. The LRPL nodes ascertained on follow-up MRI were considered positive for metastases. First, the optimal cutoff value of each parameter was derived for each parameter. In addition, neural network (NN) nodal evaluation was tested for all combinations of three parameters, namely MIAD, maximal axial diameter (MAAD), and maximal coronal diameter (MACD). The optimal approach was determined through brute force attack, and the results of two methods were compared using a bootstrap sampling method. Second, the mean standard uptake value (NSUVmean) was added as the fourth parameter and tested in the same manner for 410 nodes in 219 patients.

**Results:**

In first and second analysis, the accuracy rate (percentage) for the MIAD was 89.0% (590/663) and 89.0% (365/410), with the optimal cutoff values being 6.1 mm and 6.0 mm, respectively. With the combination of all three and four parameters, the accuracy rate of the NN was 89% (288/332) and 88.8% (182/205), respectively. In prediction, the optimal combinations of the three and four parameters resulted in correct identification of three (accuracy: 593/663, 89.4%) and six additional nodes (371/410, 90.5%), representing 4% (3/73) and 13.3% (6/45) decreases in incorrect prediction, respectively.

**Conclusion:**

NPC LRPL nodes with an MIAD ≥ 6.1 mm are positive. Among nodes with an MIAD < 6.1 mm, if the NSUVmean ≥ 2.6 or MACD ≥ 25 mm and MAAD ≥ 8 mm, the nodes are positive; otherwise, they are negative.

## Introduction

Identification of positive lateral retropharyngeal lymph (LRPL) nodes in nasopharyngeal carcinoma (NPC) is crucial. However, complete nodal dissection for LRPL nodes is not feasible for newly diagnosed patients with NPC, who should be administered radiotherapy (RT) and chemotherapy as soon as tissue proof for primary cancer and the clinical stage are established. Thus, physicians must solely depend on studies of images to determine the required RT dose for an individual LRPL node and consider systemic chemotherapy according to the subsequent nodal stage. The imaging modality of choice for NPC is magnetic resonance imaging (MRI) [1]. There remains little debate in literature regarding typical findings of MRI, such as central necrosis, extracapsular invasion, and asymmetric grouping of equivocal nodes, for considering LRPL nodes as positive [1–3]. For node size, most physicians now use minimal axial diameter (MIAD) in MRI to evaluate nodal metastasis [4, 5]. The criterion of MIAD gradually shifted from 5 to 6 mm after Zhang reported that these criteria have accuracies of 85.8% and 87.5%, respectively. The data were reviewed and the methodology was deemed to be standard and robust [2]. After wide consideration of 6 mm as the new criterion for diagnosing LRPL nodes, no paper has discussed the diagnostic role of any other nodal diameters in MRI. Other measurable nodal diameters include maximal axial diameter (MAAD) and maximal coronal diameter (MACD), which have been assessed mainly for evaluating the nodal responsiveness to anticancer treatment [6, 7]. MAAD was deemed to be inferior because of its obviously lower area under the curve (AUC) of the receiver operating characteristic (ROC) curve than that of MIAD [6]. The literature lacks discussion about the role of MACD. Fluorodeoxyglucose positron emission tomography–computed tomography (PET/CT) is very effective for diagnosing cervical lymph nodal metastasis in head and neck cancer as well as in NPC [8–11]. PET/CT also facilitates accurately predicting distant metastasis by evaluating measured nodal data [12]. As a functional imaging tool, PET/CT can be used in conjunction with MRI to improve results [13]. However, the net additional contribution of PET/CT used to complement MRI has not been addressed in the literature on LRPL nodal metastasis in NPC. For equivocal LRPL nodes in NPC patients, whose chemoradiation decisions can solely be based on image information in the absence of feasible tissue proof, it is imperative for improving the accuracy of node diagnosis [2, 5, 10, 11, 13, 14]. In stage I, this paper describes an evaluation of nodal parameters by using a neural network (NN) and an exhaustive key search of 663 LRPL nodes in NPC for testing whether higher accuracy than that of MIAD alone can be attained by employing a combination of MIAD, MAAD, and MACD. In addition, the optimal cutoff value for each parameter was determined. In stage II, 410 nodes were jointly evaluated using the method applied in stage I to determine whether superior results could be attained by adding the nodal mean standard uptake value (NSUVmean) as a parameter.

## Materials and Methods

Name of the IRB: Institutional Review Board of the Chi Mei Medical Center Approval numbers: 10111-L03 and 10508-L04. Although consent was not specifically obtained for this retrospective review, all information was anonymized and deidentified prior to its analysis.

### Patients and lymph nodes

Previously, we collected LRPL nodes with their nature known according to RT responsiveness and long-term image follow-up data [5, 15].

**Fig 1:**
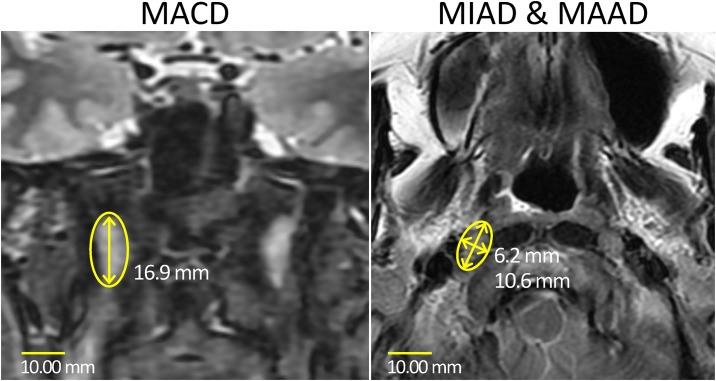
Measuring the three diameters of a right lateral retropharyngeal node. This image was obtained from a 48-year-old NPC woman with a lateral retropharyngeal lymph node (yellow circled) on a T2-weighted MRI. (a) In axial plane, the MAAD (10.6 mm) should be measured first, and the MIAD (6.2 mm) should then be measured in the perpendicular direction. (b) Coronal plane of image showing that the MACD (16.9 mm) of the node can be measured using an electronic caliper easily. NPC, nasopharyngeal cancer; MIAD, minimal axial diameter; MAAD, maximal axial diameter; MACD, maximal coronal diameter.

Institutional review boards approved the analyses of 335 eligible NPC patients with 663 LRPL nodes. Although consent was not specifically obtained for this retrospective review, all information was anonymized and deidentified prior to its analysis. All image data acquisition protocols were similar and were previously published [5, 15]. A relatively large (MIAD up to 10 mm) necrotic node, which did not promptly respond to RT, was excluded to facilitate attaining true diagnostic criteria identical to those of a Chinese group. Thus, 141 LRPL nodes were chosen from 71 Taiwanese NPC patients and 522 LRPL nodes were chosen from 264 Chinese NPC cases after excluding a medial retropharyngeal node. The MIAD and MAAD as well as the MACD (Fig 1) were measured. Among the LRPL nodes in the NPC patients, only 410 nodes in 219 patients had data on the NSUVmean. All nodes lacking NSUV data were from Chinese patients. The imaging protocol for the NSUVmean was similar for the two countries and has been described in previous publications [15, 16]. In the Chinese patients, PET/CT data were qualitatively categorized only for negative nodes, whereas the NSUVmean of lesions with abnormally high uptake was measured by applying an ROI to fit those lesions. For the Taiwanese patients, the NSUVmean was measured using an ROI circle with a diameter equal in size to the nodal MIAD.

### Neural network

A feed-forward back-propagation NN was established in MATLAB (R2013a). This NN comprised one hidden layer with a tansig operator and an output layer with a linear operator. This network setup is generally adopted for classification. At first, three parameters, MIAD, MAAD, and MACD (stage I), and the confirmed diagnoses of the nodes were included in the input data of the network. We optimized the diagnosis of LRPL nodes by minimizing the mean square error between the calculated output after iterations and the desired output.

By selection different number within the three parameters, seven combinations were used as the input variable(s). Roughly half, 331, of the LRPL nodes were randomly selected as the NN training data set. (There was not validation data set.) The remaining 332 nodes were used to test the performance of the NN model. In addition, the accuracy, specificity, sensitivity, positive predictive value, and negative predictive value were derived. Later, the NSUVmean was added (stage II) for an identical NN evaluation of 410 nodes, with a training group of 205 nodes and a testing group of 205 nodes. For the 120 Chinese nodes of which the quantitative NSUVmean was not measured because of an apparently negative classification, we assumed their NSUVmean to be zero. Before the evaluation, the NSUVmean was added with a unit to serve as input data for NN training and testing. The NN results were compared with expert evaluations only for the MIAD and NSUVmean parameters because there are no widely recommended criteria for the MAAD and MACD in the literature regarding LRPL nodes in NPC. After the optimal cutoff values of the MAAD and MACD were provided for human evaluation, 7 and 15 combinations of three and four parameters, respectively, were employed in comparing the NN versus expert evaluation. This evaluation was conducted blindly for 141 LRPL nodes from Taiwanese NPC patients by a senior physician experienced in NPC diagnosis and with board certification in diagnostic radiology in Taiwan.

### Diagnostic efficacy evaluation and the optimal cutoff values of diameters

Both Youden’s index (specificity + sensitivity– 1) [17] and the accuracy were used to assess the optimal cutoff value according to the data of each parameter. ROC curves were used for evaluating the effectiveness of the prediction of each nodal diameter. The optimal cutoff value of each parameter was derived according to Youden’s index and accuracy. The optimal approach was identified through an exhaustive key search. These procedures were performed for stage I and stage II.

### Brute force attack

Prediction accuracy was calculated using the formula of the consistency rate (number of consistent predictions divided by the total number) under a specific condition where the cutoff point of MIAD was determined prior to those of MAAD and MACD. Two formulas were applied to two alternative formats of measures. Specifically, the following two formulas with either “and” or “or” representing the condition of MIAD and MAAD with respect to the given MIAD condition were used in Microsoft Excel: (i) IF(MIAD > MIAD cutoff point,1,IF(AND(MAAD > MAAD cutoff point, MACD > MACD cutoff point),1,0)), and (ii) IF(MIAD > MIAD cutoff point,1,IF(OR(MAAD > MAAD cutoff point, MACD > MACD cutoff point),1,0)). Brute force attack, an exhaustive key search for each possible cutoff point, was used in this study to determine the cutoff points [18]. The significance of the difference in the accuracy of the MIAD and the optimal approach was evaluated according to the 95% confidence interval (95%CI) by using a bootstrap sampling method [19]. These procedures were also performed for stage I and stage II.

### Statistical analysis

ROC curves were calculated using SPSS for Windows version 12 (SPSS, Chicago, IL). The exhaustive key search was performed using Microsoft Excel. Bootstrap sampling was performed using the MedCalc software for Windows, version 9.5.0.0 (MedCalc Software, Mariakerke, Belgium). All curves in figures were confirmed using Excel 2007 version 5.2.10 (Microsoft, Redmond, WA). Again, these procedures were performed for both stage I and stage II.

## Results

### Patients, lymph nodes, and the predictive power of each factor

**Fig 2:**
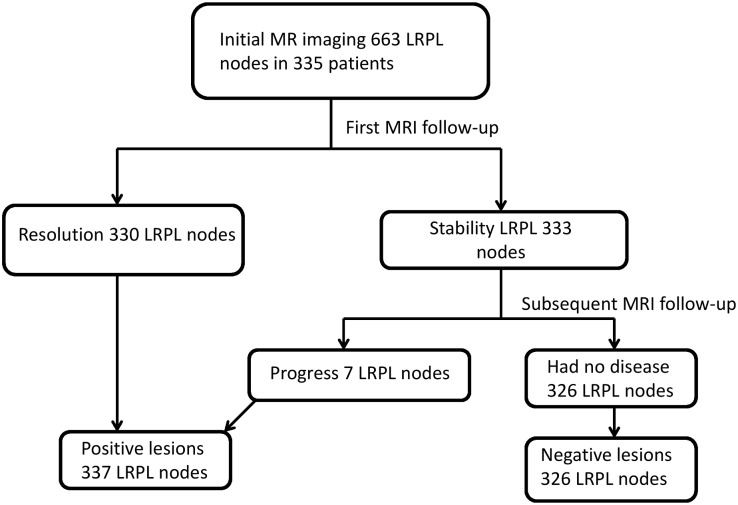
Stage I flowchart of MRI follow-up. Diagnostic results of MRI follow-up for 663 nodes in 335 patients with data on three diameters from MRI.

**Fig 3:**
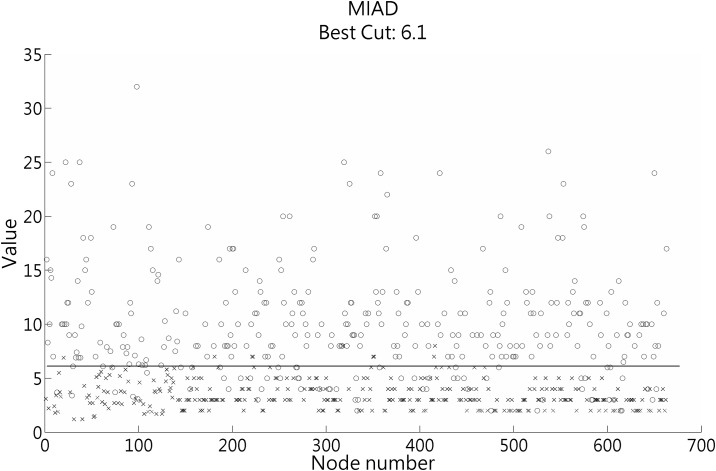
Scatter plots of the MIAD in stage I. Scatter plots of the minimal axial diameter (MIAD) for positive nodes (○) and negative nodes (×) with their optimal cutoff value shown by the line.

**Fig 4:**
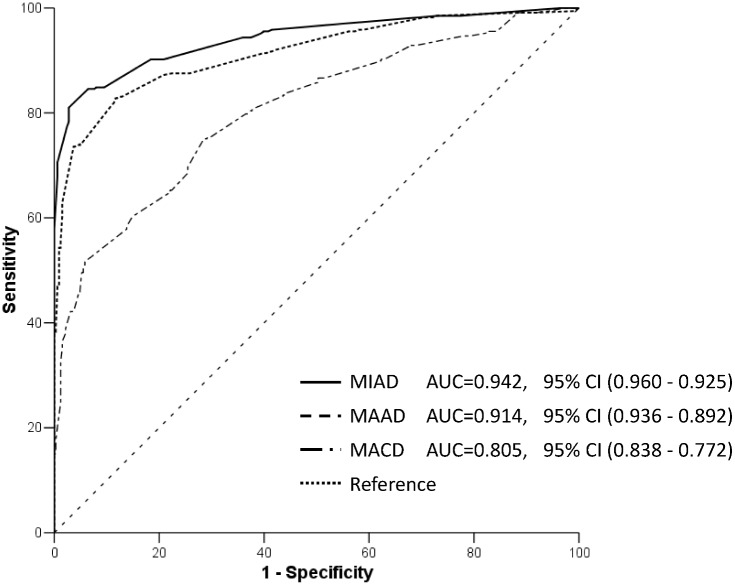
ROC curves of parameters in stage I. Receiver operating characteristic curves of the three nodal diameters: minimal axial diameter (MIAD), maximal axial diameter (MAAD), and maximal coronal diameter (MACD). The MACD is a much less valid criterion because the area under the curve is obviously lower than that of the other two diameters (without any 95% confidence interval overlapping of its area under curve to that of MIAD and MAAD).

**Fig 5:**
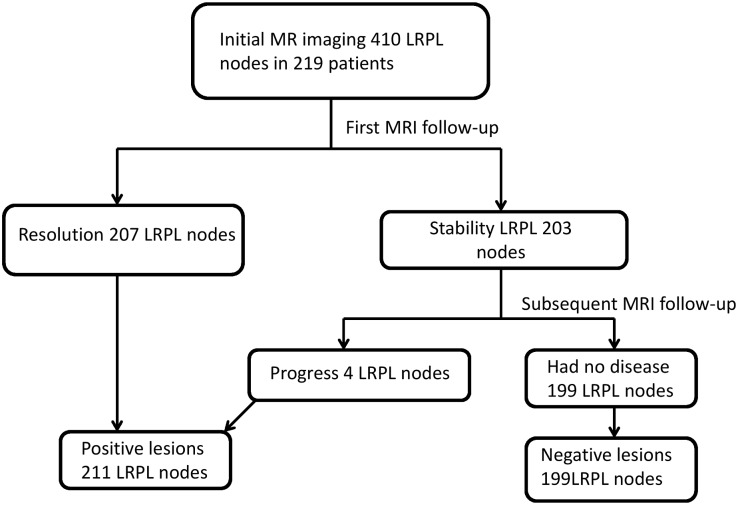
Stage II flowchart of MRI follow-up. Diagnostic results of MRI follow-up for 410 nodes in 219 patients with data on four parameters from MRI and PET/CT.

**Fig 6:**
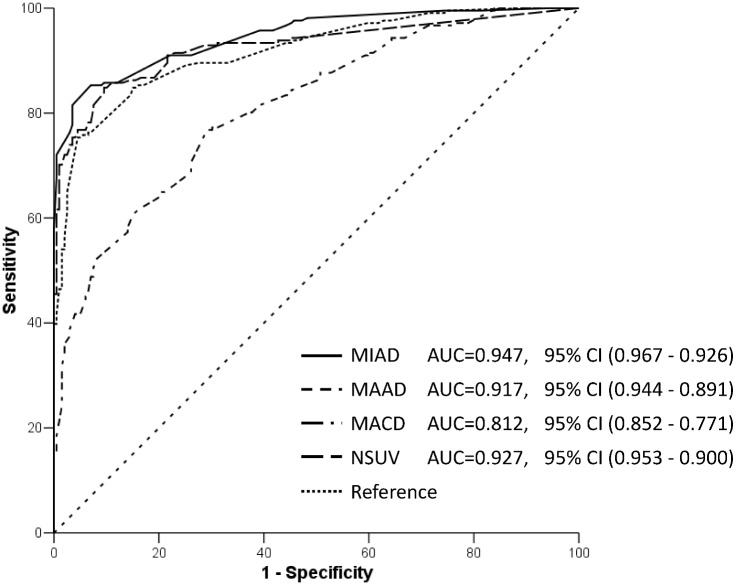
ROC curves of parameters in stage II. ROC curves of the three nodal diameters: MIAD, MAAD, MACD, and NSUV. Some overlap of 95%CIs of the area under the curves of the MIAD, MAAD, and NSUV is evident. MIAD, minimal axial diameter; MAAD, maximal axial diameter; MACD, maximal coronal diameter.

The 335 patients had 663 LRPL nodes in stage I; specifically, the 71 Taiwanese patients had 141 nodes and the 149 Chinese patients had 522 nodes. On the basis of the results at MRI follow-up, we determined 337 of 523 (50.8%) LRPL nodes to be positive, whereas the other 326 nodes were classified as negative (Fig 2). A scatter plot of MIAD with its optimal cutoff value is shown in (Fig 3). The optimal cutoff value, accuracy, and Youden’s index for MIAD were 6.1 mm, 89.1% (590/663), and 78.3%, respectively; for MAAD, these values were 8.4 mm, 85.52% (567/663), and 71.1%, respectively; and for MCAD, 15.9 mm, 73.3% (486/663), and 46.5%, respectively. The ROC curves of the three nodal diameters are shown in (Fig 4). MIAD had the highest AUC of 0.942. In stage II evaluation, 219 patients had 410 visible nodes. Of the LRPL nodes, 211 were classified as positive and 199 were classified as negative (Fig 5). The optimal cutoff values (nodes ≥ value as positive), accuracy, and Youden’s index were 6.0 mm, 89.0% (365/410), and 78.3% for the MIAD; 10.0 mm, 85.1% (349/410), and 70.8% for the MAAD; 16.0 mm, 73.9% (303/410), and 47.6% for the MCAD; and 1.76, 87.6% (359/410), and 75.3% for the NSUVmean, respectively. The ROC curve for each parameter is shown in Fig 6.

### Comparisons of NN versus expert evaluation

For stage I evaluation, the accuracy, sensitivity, specificity, positive predictive value, and negative predictive value of the NN were 89% (294/332), 84% (134/159), 92% (160/173), 91% (134/147), and 86% (160/185), respectively. The accuracy rates for all combinations of the three parameters are shown in Table 1. For MIAD alone, the accuracy rates determined by the NN and expert were 90.4% (300/332) and 86.5% (122/141), respectively.

**Table 1:** Results of NN and expert evaluation for stage I.

Parameters	NN Accuracy(%) in 332 nodes	Expert Accuracy(%) in141 nodes
MIAD + MAAD + MACD	294(88.6%)	122(86.6%)
MIAD +MAAD	296(89.2%)	126(89.4%)
MIAD +MACD	297(88.9%)	121(85.8%)
MAAD +MACD	282(84.9%)	120(85.1%)
MIAD	300(90.4%)	122(86.5%)
MAAD	291(87.7%)	125(88.7%)
MACD	238(71.7%)	100(70.9%)

Accuracy of the NN and expert evaluation for seven combinations of three parameters in 332 nodes. Note: We provided the optimal cutoff value of the MAAD and MACD for experts to conduct evaluation.

NN, neural network; MIAD, minimal axial diameter; MAAD, maximal axial diameter; MACD, maximal coronal diameter.

In stage II, the accuracy of the NN was 88.8% (182/205), whereas that of expert evaluation was 80.9% (114/141). The accuracy for all combinations in stage II is listed in Tables 2 and 3.

**Table 2:** Accuracy, sensitivity, specificity, and positive and negative predictive values of the NN and expert evaluation in the stage II comparison.

	Accuracy (%)	Sensitivity (%)	Specificity (%)	PPV (%)	NPV (%)
NN test 205 nodes	182/205(88.8%)	83/101(82.2%)	99/104(95.2%)	83/88(94.3%)	99/117(84.6%)
Human 141 nodes	114/141(80.9%)	70/74(94.6%)	44/67(65.7%)	70/93(75.3%)	44/48(91.7%)

Results of the comparison between the NN and expert evaluation in four parameters, namely the MIAD, MAAD, MACD, and NSUVmean. Note: The accuracy of the NN was 7.9% higher than that of expert evaluation. Although expert evaluation had higher sensitivity, the specificity was excessively low.

NN, neural network; PPV, positive predictive value; NPV, negative predictive value.

**Table 3:** Accuracy comparison of the NN for 205 nodes and expert evaluation for 141 nodes for 15 combinations of four parameters.

Parameters	NN accuracy (%)	Human accuracy (%)
A1A2CN	182(88.8%)	114(80.9%)
A1A2C	174(84.9%)	122(86.5%)
A1A2N	176(85.9%)	114(80.9%)
A1CN	176(85.9%)	110(78.0%)
A2CN	185(89.8%)	113(80.1%)
A1A2	184(89.8%)	126(89.4%)
A1C	178(86.8%)	121(85.8%)
A1N	186(90.7%)	108(76.6%)
CN	180(87.8%)	110(78.0%)
A2C	176(85.9%)	120(85.1%)
A2N	188(91.7%)	112(79.4%)
A1	184(89.8%)	122(86.5%)
A2	174(84.9%)	125(88.7%)
C	153(74.6%)	115(81.6%)
N	174(84.9%)	100(70.9%)

In the stage II comparison, we observed that the NN results were more consistent and accurate than those of expert evaluation for most combinations.

NN, neural network; A1, maximal axial diameter: A2, maximal axial diameter; C, maximal coronal diameter; N, nodal mean standard uptake value.

### Results of brute force attack

**Fig 7:**
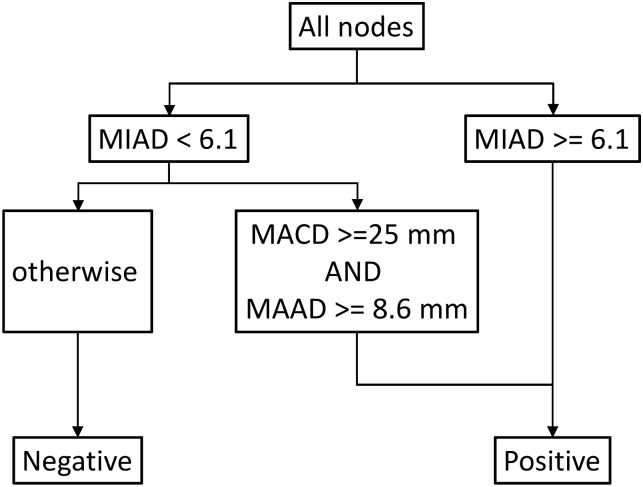
Stage I optimal approach. With the method of brute force attack, nodes were determined to be positive according to an MIAD ≥ 6.1 mm. Note that the combination approach increased the accuracy by only 0.4% [from 89.1% (590/663) to 89.4% (593/663)] by correcting three false negative errors. MIAD, minimal axial diameter; MAAD, maximal axial diameter; MACD, maximal coronal diameter.

In stage I brute force attack, we assessed the optimal approach, including nodes that were determined to be positive according to MIAD ≥ 6.1 mm. Among nodes with an MIAD smaller than 6.1 mm, if MACD ≥ 25 mm and MAAD ≥ 8.4 mm, the nodes are still positive; otherwise, the nodes were considered negative (Fig 7). With this approach, the diagnosis of three additional nodes was achieved and 4% (3/73) decreasing in wrong prediction noted, with the total accuracy being 89.4% (593/663) compared with the 89.0% (590/663) obtained using only the MIAD cutoff of 6.1 mm. Combining only MACD and MIAD provided the accurate diagnosis of only one additional node.

**Fig 8:**
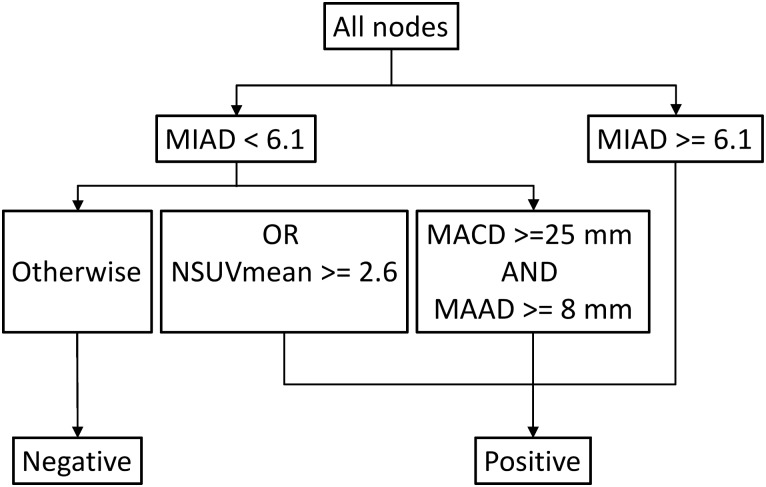
Stage II optimal approach. The proposed approach determined through brute force attack: The combination of the MIAD, MAAD, MACD, and NSUV prevented the false positive diagnosis of as many as 6 in 45 nodes (13.3%). MIAD, minimal axial diameter; MAAD, maximal axial diameter; MACD, maximal coronal diameter; NSUVmean, mean standard uptake value.

**Fig 9:**
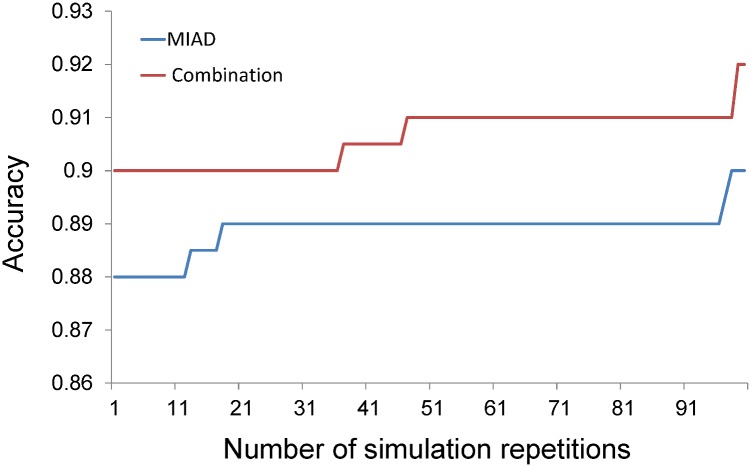
Bootstrap sampling method for comparing the stage II parameter combination versus the MIAD alone. Comparison of the combination method and the MIAD alone by sampling 100 cases and simulating 100 repetitions to obtain the median of accuracy and plot the line chart (if the sampling data have a Gaussian distribution, then the median can be replaced by the mean; thus, t = 42.57 and p < 10^−64^). MIAD, minimal axial diameter; MAAD, maximal axial diameter; MACD, maximal coronal diameter; NSUVmean, nodal mean standard uptake value.

In stage II brute force attack, NPC LRPL nodes with an MIAD ≥ 6.1 mm were positive. If the NSUVmean ≥ 2.6 or if the MACD ≥ 25 mm and MAAD ≥ 8 mm, nodes with an MIAD < 6.1 mm were positive; otherwise, they were negative (Fig 8). The accuracy of this diagnostic combination was 90.5% (371/410), and the false negative diagnoses of six nodes were corrected, representing an error reduction rate of 13.3% (6/45); by contrast, using only the MIAD with a cutoff of 6.0 mm yielded an accuracy of 89.0% (365/410). The 95%CIs of the two methods (90.0% to 92.0% and 88.0% to 90.0%) overlapped at only one point of 90% (Fig 9). The difference in accuracy was at least marginally statistically significant (p < 0.06). When we replaced the median with the mean, the data exhibited a Gaussian distribution, with t = 42.57 and p < 10^−64^.

## Discussion

The pooled NPC patients with LRPL nodes from two countries had similar distributions of epidemiologic characteristics, WHO histological grading, clinical stages, and imaging protocols [5, 15]. These similarities and an identical definition of true diagnosis were imperative for our analysis. Because the accuracy rate of the criterion proposed by Zhang [5] is high, further improvement is not easy. In this study, 141 Taiwanese LRPL nodes were added for analysis and a single MIAD cutoff at 6.1 mm achieved a slightly higher accuracy rate of 89.0% (590/663) compared with the accuracy of 87.5% and 85.8% achieved for the 6- and 5-mm cutoffs, respectively, reported by Zhang. Before the publication of Zhang’s paper in 2010, MIAD ≥5 mm was the most commonly used criterion for positive identification in relevant studies [4, 10, 14, 20–22]. Some physicians might hesitate to accept the change of the criterion standard promptly because of the limitations listed in Zhang’s paper [5] and the slow progress of subsequent studies in verifying the proposed radiologic criterion. One study used similar diagnostic criteria for identifying cancerous nodes and claimed the optimal cutoff value of the MIAD to be 4.5 mm [23]. Currently, no other new articles support this cutoff value, and we identified some problems indicating that this cutoff is invalid; the extraordinarily high recurrence rate of stable nodes after RT within a relatively short mean follow-up time; the unreasonably low accuracy and sensitivity of the MIAD cutoff; some nodes unusual categorization as positive only because they lost their contrast enhancement after RT. In summary, we do not believe the optimal MIAD cutoff value of 4.5 mm to be reasonable.

By contrast, Zhang’s methodology was confirmed to be robust and involved systemic standard evaluation [2]. Moreover, a recent article further validated 6 mm as a superior cutoff value according to overall survival and distant metastasis-free survival data [3]. The optimal single MIAD cutoff value of 6.1 mm determined according to our data is compatible with the value proposed by Zhang. This value can be refined to the submillimeter level, which is consequential because of the convenient measurement of the node size (Fig 1) by using electronic calipers according to the World Health Organization criteria in the magnified T2-weighted images of hospital imaging systems [7]. Thus, we deduced that identifying a value in decimals would be practical. In addition, when derived according to Youden’s index, the optimal cutoff value of MIAD was 6.1 mm. This index emphasizes the balance between sensitivity and specificity. The precondition of using Youden’s index is equal damage from false-negative and false-positive cases according to judgment. We presume that this premise applies to our clinical cases. Both accuracy and Youden’s index indicated the same cutoff value of 6.1 mm, thus validating the conclusion of Zhang.

We determined the NN to be very effective because it provided accurate results at least comparable to those of the results by Zhang, the expert evaluation, and the brute force attack. Theoretically, the NN is a robust tool in treating multiple complexly (nonlinear) dependent or interrelated parameters for dichromatic classification. However, the NN failed to achieve a significantly superior result for the stage I and stage II combinations of parameters compared with the MIAD (Tables 1 and 3). We thus inferred that MIAD alone is sufficient for diagnosing LRPL nodes in NPC. After the brute force attack, marginal (only 0.4%) improvements in accuracy were observed after the other nodal parameters were added. In diagnostic practice, the MIAD—instead of other diameters—pivotally determines the roundness of node with a tendency toward metastasis. It is much more parsimonious to use a single parameter than to use a combination of three parameters in practice. The negative findings in stage I are vital for both therapeutic and diagnostic radiologists who require evidence to show that presently MIAD is the optimal imaging diagnostic criteria for LRPL node metastasis in NPC. The results of the bootstrap sampling method [19] revealed that the combination approach was significantly superior to the MIAD (*p* < 0.0001; figure in S1 Appendix) and had robust performance when the data exhibited a Gaussian distribution. The median of the results was replaced with the mean for an additional t test; however, because the improvement in accuracy was only 0.4%, we employed the median instead of the mean to evaluate the results of bootstrap sampling. The 95%CI of 100 randomly selected simulations revealed that the median overlapped in the range of 89.0% to 89.25%, with p > 0.05. Moreover, the bootstrap method may have the limitation of overfitting, for which a potential solution must be developed to validate the combination approach in an external cohort in the future.

In stage II, although the NN 7.9% higher accuracy than that of expert evaluation for four-parameter combinations (Table 2), the NN failed to achieve a significantly superior result through the combination of four parameters compared with the MIAD alone (Table 3).

However, brute force attack in stage II showed that the combination of four parameters (Fig 8) prevented the false negative diagnosis of as many as 6 in 45 nodes (13.3%). The reason why the NN failed to attain appreciably superior results by using the combination approach remains unclear, because the underlying mechanisms of the NN are difficult to determine. We postulate two reasons. First, the NN used only half of the nodes (205) for training. Second, the optimal cutoff value for the NSUVmean was 1.76, which is reasonably far from the criterion used for brute force attack (NSUVmean ≥ 2.6 indicates positivity if MIAD < 6.1 mm).

Under the new standard, the criterion for LRPL nodal metastasis is MIAD ≥ 6.1 mm; using this criterion in combination with other factors can correct 6 false negative diagnoses compared with using only the MIAD parameter with an optimal cutoff of 6.0 mm (error reduction rate of 13.3%). The clinical significance of this criterion is that it can prevent radiation oncologists from prescribing RT doses greater than 60 Gy for negative LRPL nodes, and it is more straightforward than an MIAD ranging from 5.0 to 5.9 mm. Excessive RT doses, especially 66 to 70 Gy, cause a higher incidence of complications such as internal carotid artery injury [24] and swallowing dysfunction [25] and more severe dryness of mouth [26–28]. If a LRPL node is the only node with suspected involvement, our criteria can prevent patients from undergoing unnecessary chemotherapy.

In the past, both PET/CT and MRI were essential [13] for nodal stage evaluation in NPC patients. Equivocal LRPL nodes are usually significantly smaller than cervical nodes; accordingly, MRI was considered the diagnostic method of choice [1] because it directly visualizes these small nodes in clear soft tissue contrast with a distinct boundary indicating the nodal capsule. Inspecting Fig 3 reveals that using an MIAD value greater than 6.1 mm yielded few problems, whereas the number of false negative lesions was relatively high below this value. PET/CT can provide quantitative data on the NSUVmean, and integrating CT with PET or even MRI of the same patient within a short time interval can facilitate measuring the lesion as accurately as possible. Measuring small nodal lesions by using PET/CT entails some problems: First, the intensity of the detector count can be reduced by spill-out (partial volume) effects, which potentially require partial volume correction (PVC) [8]. However, our previous study [15] demonstrated that PVC was beneficial only for isolated LRPL nodes with an MIAD ≥ 6 mm. More complex is the spill-in effect [29–31], which can originate from the node being located near the primary tumor, lymphadenopathy, and a high NSUVmean. This is not a rare clinical scenario and resolving it is completely beyond the limit of our capability. It also was the reason why the criterion of the NSUVmean used in the combination of parameters in stage II analysis had to be as high as ≥ 2.6, which is markedly higher than the optimal cutoff value of 1.76, in the diagnostic decision system. This prevented overcorrection of originally true negative cases to become false positive.

After stage II brute force attack with 410 nodes, six false negative errors obtained by using only the MIAD with a cutoff of 6.0 mm could be corrected. We noted in stage I with 663 nodes that the MIAD alone with a cutoff of 6.1 mm had the highest accuracy; accordingly, this cutoff was employed in the proposed criteria in stage II (Fig 8). When positive nodes were identified according to an MIAD ≥ 6.1, applying the NSUVmean criterion corrected four false negative errors and the combination of MAAD and MACD criteria corrected three false negative errors. In addition, the bootstrap sampling method (Fig 9) revealed that the 95%CIs of the median values overlapped at only one point, 90.0% (p < 0.06). The marginal significant superior results achieved using the combination method. However, it still needs to be validated in an additional cohort. These reasons encourage us to draw a conclusion to be the results of the stage II brute force attack (Fig 8).

The current study had several other limitations. First, it did not involve histopathology, which may have perpetuated some diagnostic errors. Tissue proof for LRPL nodes can be obtained through image-guided needle aspiration, nodal excision, or even en-bloc resection. Use of these methods has been reported only in recurrent cases [32–35]. In the current standard of NPC treatment, it is impossible to routinely achieve complete LRPL nodal dissection to perform comprehensive pathologic evaluation before chemotherapy and RT. The definition of the true diagnosis was evaluated as the most robust method [2]. Second, the combined factors from MRI were only diameters; diffusion-weighted imaging, reportedly useful in LRPL node diagnosis, except for tiny nodes [23], was not included because of the limitations of the facility at our hospital. The use of PET/MR is now increasing in clinical practice [36–39]. For cases of early stage NPC with equivocal LRPL nodes, a highly useful biomarker for NPC detection, the Epstein–Barr virus DNA serum titer [40–42], can be incorporated into tests with the aforementioned methods and applied in future studies.

In conclusion, we propose new diagnostic criteria for LRPL nodal metastasis of NPC: NPC LRPL nodes with an MIAD ≥ 6.1 mm are positive. Among nodes with an MIAD < 6.1 mm, if the NSUVmean ≥ 2.6 or if the MACD ≥ 25 mm and MAAD ≥ 8 mm, the nodes are still positive; otherwise, they are negative.

## Supporting Information

S1 Appendix Comparison of the minimal axial diameter (MIAD) and stage I combination method by using bootstrap sampling. (DOCX)
