# Supplementary material for: Can Parameters Other than Minimal Axial Diameter in MRI and PET/CT Further Improve Diagnostic Accuracy for Equivocal Retropharyngeal Lymph Nodes in Nasopharyngeal Carcinoma?
Source: PLoS One. 2016 Oct 13;11(10):e0163741. doi: 10.1371/journal.pone.0163741 (PMC5063369; doi:10.1371/journal.pone.0163741)

Supporting information

Appendix S1

In stage I, we compared the accuracy of the combination of the nodal diameters with that of the minimal axial diameter (MIAD) alone by using the bootstrap sampling method. After repeatedly sampling 100 nodes randomly from 663 nodes to conduct each test with the derived accuracy data 100 times, we ranked the methods and plotted the accuracy data in the figure. The 95% confidence intervals (95%CIs) of the medians of the combination method and MIAD alone were 0.8900−0.8950 and 0.8850−0.8925, respectively. The 95%CIs overlapped in a trivial range of 0.0025. However, the mean difference between the two methods was significant, with a *t* value of 9.95 (*p* < 0.0001), as Figure 1 shows, indicating that the combination approach outperforms the MIAD method when cutoff points are referenced.

Figure 1: Comparison of the minimal axial diameter (MIAD) and stage I combination method by using bootstrap sampling.


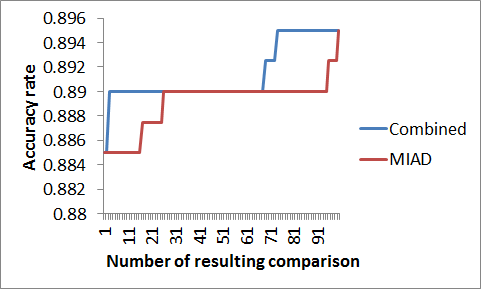

Supplement: S1 Appendix — (DOCX) [file pone.0163741.s001.docx]
